# Stable mucus-associated bacterial communities in bleached and healthy corals of *Porites lobata* from the Arabian Seas

**DOI:** 10.1038/srep45362

**Published:** 2017-03-31

**Authors:** Ghaida Hadaidi, Till Röthig, Lauren K. Yum, Maren Ziegler, Chatchanit Arif, Cornelia Roder, John Burt, Christian R. Voolstra

**Affiliations:** 1 Red Sea Research Center, Division of Biological and Environmental Science and Engineering (BESE), King Abdullah University of Science and Technology (KAUST), Thuwal, Saudi Arabia; 2 Center for Genomics and Systems Biology, New York University Abu Dhabi, PO Box 129188, Abu Dhabi, United Arab Emirates

## Abstract

Coral reefs are subject to coral bleaching manifested by the loss of endosymbiotic algae from coral host tissue. Besides algae, corals associate with bacteria. In particular, bacteria residing in the surface mucus layer are thought to mediate coral health, but their role in coral bleaching is unknown. We collected mucus from bleached and healthy *Porites lobata* colonies in the Persian/Arabian Gulf (PAG) and the Red Sea (RS) to investigate bacterial microbiome composition using 16S rRNA gene amplicon sequencing. We found that bacterial community structure was notably similar in bleached and healthy corals, and the most abundant bacterial taxa were identical. However, fine-scale differences in bacterial community composition between the PAG and RS were present and aligned with predicted differences in sulfur- and nitrogen-cycling processes. Based on our data, we argue that bleached corals benefit from the stable composition of mucus bacteria that resemble their healthy coral counterparts and presumably provide a conserved suite of protective functions, but monitoring of post-bleaching survival is needed to further confirm this assumption. Conversely, fine-scale site-specific differences highlight flexibility of the bacterial microbiome that may underlie adjustment to local environmental conditions and contribute to the widespread success of *Porites lobata*.

Corals live in an endosymbiotic relationship with photosynthetic algae of the genus *Symbiodinium*, along with other microorganisms, such as bacteria, archaea, fungi, and viruses^1^,^2^. This consortium constitutes a metaorganism commonly referred to as the coral holobiont^3^. While the algal symbionts provide a large part of the coral host’s metabolic requirements^4^, the role of coral-associated bacteria, although suggested to be functionally important, is less well understood^5^,^6^. Studies have shown that the bacterial community provides nutrients, confers protection from pathogen invasion through antimicrobial production, and is indicative of coral health states^7^,^8^,^9^,^10^,^11^,^12^,^13^,^14^.

Importantly, the coral host provides a range of habitats such as the coral tissue, skeleton, and surface mucus layer (SML) that harbor distinct and diverse bacteria, but only few studies characterized the differences in community composition and function^15^,^16^,^17^,^18^. In particular, the SML provides a protective barrier and constitutes a highly selected microbial environment critical for coral health^8^,^19^,^20^. Coral mucus is a complex mixture of carbohydrates, lipids, and proteins, and plays a fundamental role in structural support, heterotrophic feeding, sediment cleansing, defense against environmental stressors, besides a suite of other functions^20^,^21^,^22^. Coral mucus is produced in the ectodermal mucus gland cells and originates from photosynthates and compounds derived from heterotrophic feeding^23^,^24^. In many cases, the SML is a transparent coat that changes over days to mucus sheets. Aged mucus sheets are detached from the coral surface into the water column, after which new fluidic mucus is produced at the coral surface leading to a new cycle^20^,^23^. Detached coral mucus sheets are shown to play an important role in retaining and recycling nutrients and metabolites in coral reef ecosystems^25^.

Anthropogenic impacts in the form of local (e.g., overfishing and nutrient enrichment) and global (e.g., ocean warming and ocean acidification) stressors have resulted in a substantial loss of coral cover over the last decades, manifested by an increase in coral bleaching and coral disease^26^,^27^,^28^. Coral bleaching describes the physical whitening of the coral colony due to loss of its pigmented algal symbionts and can be induced by different stressors, of which climate changed-induced global warming is the most threatening^29^. Yet, the molecular mechanisms behind coral bleaching are still not completely understood. Previous studies have shown that bacteria can cause coral bleaching and demonstrated that coral bleaching is related to the presence of the bacterium *Vibrio shilonii* in the annual bleaching of the Mediterranean coral *Oculina patagonica* during warm summer months^30^, but not all studies found *Vibrio shilonii* in bleached corals^31^. More generally, studies have found differences in bacterial assemblages between bleached and healthy corals indicating that bacteria respond to coral bleaching, although the precise role of bacteria in coral health and coral bleaching is not well understood^2^,^17^,^32^.

Given the worldwide increase in coral bleaching and the projected increase in frequency of global mass bleaching events^33^, it is crucial to better understand the contribution of bacteria to coral bleaching. In particular, analysis of bacteria associated with the SML might provide further insight, given that the SML provides a first protective barrier of the coral host against invading microbes. However, only few studies have analyzed coral mucus-associated bacterial communities in coral bleaching. In addition, the effect of coral bleaching on bacterial communities of corals from different locations is virtually unknown. In particular, studies analyzing bacterial communities of corals from the PAG and RS might be highly informative in this regard, as both regions display high water temperatures and are considered unique environments where corals thrive under extreme environmental conditions. Studying these potential coral refugia^34^,^35^ may provide a model for understanding the effects of global environmental change^36^,^37^,^38^.

In this study, we analyzed bacterial communities of the SML from bleached and healthy coral colonies of *Porites lobata* that were collected in the PAG and the central RS using 16S rRNA gene amplicon sequencing. Our aim was to document bacterial community composition and potential bacterial shifts between bleached and healthy corals to further understand the role of SML-associated bacteria in coral bleaching. To our knowledge, this is the first comparison of mucus-associated bacterial communities of bleached and healthy corals from the PAG and RS.

## Results

### Algal symbionts associated with *Porites lobata*

To reveal potential correlations between bacterial communities and algal symbionts associated with mucus from *P. lobata* from the PAG and RS, we conducted ITS2 DGGE-fingerprinting and subsequent sequencing of prominent bands. Our analysis revealed that *P. lobata* from the PAG were exclusively associated with *Symbiodinium* type C3 (Supplementary Table S1). In comparison, colonies from the RS were associated with *Symbiodinium* type C15 and C15 variants (C15h, C15n, C97), but we also found *Symbiodinium* types from clade D (D1, D1a, D6) in a considerable number of coral colonies (41%; Supplementary Table S1). In addition, one sample harbored *Symbiodinium* type A1. Overall, we did not detect differences in symbiont types between health states, but symbiont assemblages were different between the PAG and RS and were more variable among corals from the RS. It should be noted, however, that coral colonies were sampled based on visual inspection, hence, we cannot positively exclude that some healthy samples were affected by temperature stress, but did not yet show visual signs of paling. Further, due to difficulties in amplifying *Symbiodinium* DNA from some mucus samples (primarily bleached samples), ITS2 types could not be determined for all samples (i.e., for about a third of all coral samples).

### Bacterial community composition of coral mucus and seawater

We produced 55 16S rRNA gene libraries totaling 4,421,127 sequences from 50 *P. lobata* mucus samples (five bleached and five healthy colonies from each of two reefs of the PAG and three reefs of the RS) and five water samples (one water sample from each reef) (Table 1). After quality filtering, 2,840,780 sequences with an average length of 292 bp were retained.

**Figure 1:**
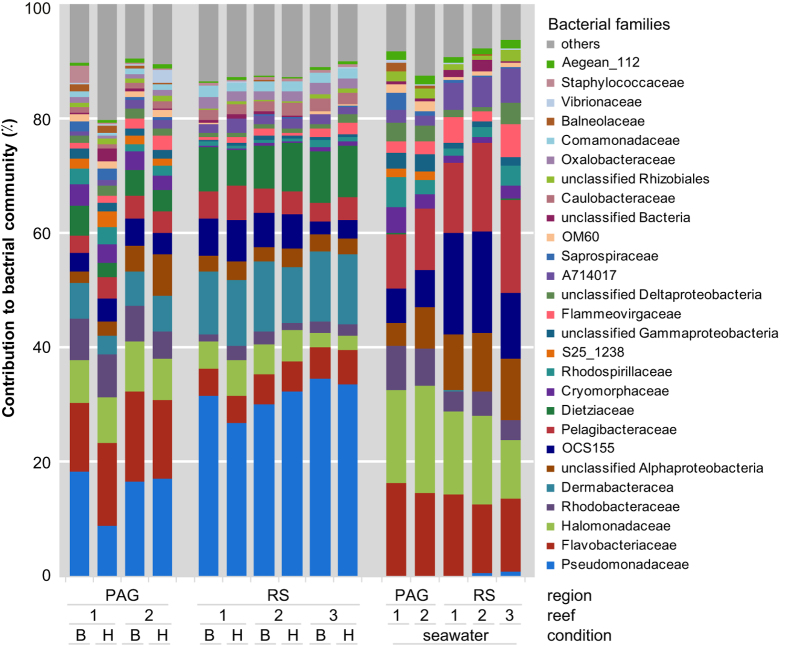
Bacterial community composition of mucus from bleached and healthy coral colonies of *P. lobata* from the Persian/Arabian Gulf (PAG) and the Red Sea (RS). Depicted is a taxonomy stacked column plot on the phylogenetic level of family. Each color represents one of the 27 most abundant families. Remaining taxa are grouped under category ‘others’. Samples are ordered by site, reef, and health-state. Values displayed as means of n = 5 for corals and n = 1 for water samples, B: Bleached; H: Healthy, PAG reef1: Saadiyat; PAG reef 2: Ras Ghanada; RS reef1: Shib Nazar; RS reef 2: Al Fahal; RS reef3: Inner Fsar.

To assess overall differences in bacterial community composition, sequences were classified to the family level (Fig. 1; Supplementary Fig. S1). There was no apparent difference between bleached and healthy coral colonies from either the PAG or RS. Rather, mucus samples from the PAG and RS appeared to be composed of the same bacterial families, but with varying degrees of abundance. For instance, corals from the PAG and RS were both dominated by bacteria from the family Pseudomonadaceae (~5% to 47%), Dermabacteraceae (~5% to 18%), and Flavobacteraceae (~3% to 19%). The former two families were particularly abundant in samples from the RS, whereas the latter one in samples from the PAG. Water samples from the PAG and RS appeared highly similar and were dominated by Flavobacteriaceae (~12% to 16%), Halomonadaceae (~10% to 18%), and Pelagibacteraceae (~9% to 16%), and markedly different from mucus samples (Fig. 1; Supplementary Fig. S1).

### Bacterial community differences of coral mucus across regions

Besides the overall similarity of bacterial community composition of mucus from *P. lobata* colonies, we were interested to assess fine-scale differences in bacterial community composition across health states (i.e., bleached and healthy) and across regions (i.e., PAG and RS). For this purpose, sequences were clustered in to OTUs after subsampling to 2,827 sequences per sample (Supplementary Dataset S1).

Species richness (Chao1) and bacterial diversity (Inverse Simpson) were highest in seawater (Table 1), but bacterial communities were not significantly different between PAG and RS (*F*s = 5.09, *P*_AMOVA _ ≥ 0.05), although a separation by region was noticeable (Supplementary Fig. S2). Conversely, water samples from the PAG and RS were significantly different from all coral mucus samples (*F*s = 18.42, *P*_AMOVA_ < 0.001). Seawater samples were excluded from subsequent analyses to emphasize on fine-scale differences of coral mucus-associated bacterial communities.

**Figure 2:**
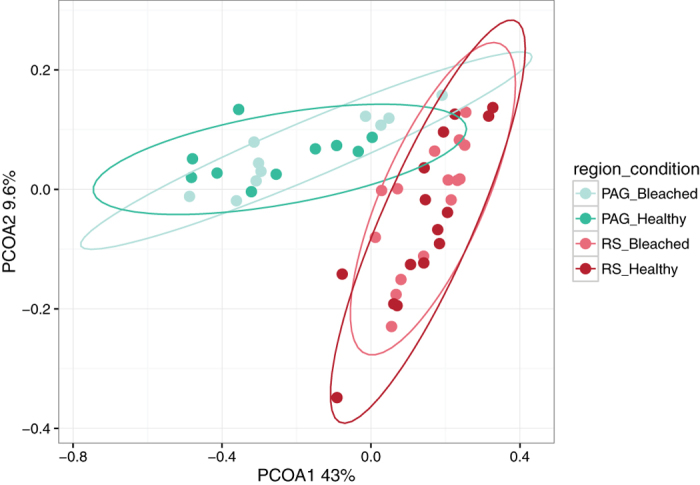
Bacterial community composition from coral mucus of bleached and healthy *Porites lobata* colonies from the Persian/Arabian Gulf (PAG) and the Red Sea (RS). Principal coordinate analysis based on Operational Taxonomic Unit (OTU) abundance (sequence counts) shows a partitioning of mucus microbiomes by region, but not by health state. Ellipses denote 95% confidence intervals per group.

Species richness as well as Simpson evenness and Inverse Simpson Indices (diversity measures) in mucus samples were on average higher for coral bacterial communities from the PAG in comparison to the RS (all *P*_t-test_ ≤ 0.001) pointing to a more diverse and heterogeneous bacterial community in coral mucus from the PAG (Table 1, Supplementary Table S2). Importantly, we did not find significant differences between bleached and healthy corals, irrespective of region (Fig. 2, *F*s = 0.92, *P*_AMOVA_ = 0.35 PAG; *F*s = 0.61, *P*_AMOVA_ = 0.72 RS). Following this, we assessed differences between all samples from the PAG and all samples from the RS and found significant differences between mucus-associated bacteria (Fig. 2, *F*s = 26.03, *P*_AMOVA_ < 0.001).

To further analyze this, we tested for indicator bacterial taxa that are significantly associated with coral mucus from either region. We found 70 bacterial OTUs that were significantly associated with mucus samples from the PAG and 24 OTUs that were significantly associated with mucus samples from the RS regardless of health state (both *P* ≤ 0.01) (Supplementary Table S3). Notably, these regional indicator taxa were mostly low abundant in the mucus samples (Supplementary Table S3) in comparison to core microbiome members (see below, Table 2).

We also tested for bacterial taxa significantly associated with health state for each region and across regions. We found 3 and 5 bacterial taxa that were significantly associated with bleached and healthy mucus samples from PAG, respectively (Supplementary Table S4). In comparison, only 1 bacterial taxon was significantly associated with healthy samples from RS, and we did not find any bacteria significantly associated with bleached samples in the RS.

### Core microbiome of coral mucus

Despite the site-specific differences in bacterial communities from the PAG and RS, we were interested in determining how many and which OTUs were consistently associated with coral mucus of *P. lobata*, as indicated by the bacterial community composition analysis (Fig. 1; Supplementary Fig. S1). To do this, we determined all OTUs that were present in at least 75% of all mucus samples and considered them members of the core microbiome. Following these criteria, we identified 42 core OTUs in mucus from *P. lobata* colonies, 6 of which were only present in mucus samples, while another 10 were primarily found in mucus. The remaining 26 core OTUs were consistently associated with coral mucus, although more abundant in seawater (Table 2). Importantly, 5 of the 42 core OTUs constituted the most abundant bacterial taxa. This suggests that despite differences in mucus-associated bacterial communities of corals from the PAG and RS, the core microbiome consistently encompasses the most abundant bacteria shared between bleached and healthy coral mucus samples, irrespective of site. Notably, *Pseudomonas veronii* (OTU0001), *Brachybacterium* sp. (OTU0002), and *Dietzia* sp. (OTU0003) that constituted the most abundant taxa are commonly associated with saline environments^39^,^40^,^41^ and corals^41^. Interestingly, and in line with Ainsworth *et al*.^42^, the 6 core taxa exclusive to mucus, i.e. *Herbaspirillum* sp. (OTU0012), *Brevibacterium aureum* (OTU0018), *Sphingomonas echinoides* (OTU0029), *Delftia* sp. (OTU0063), *Sphingobium yanoikuyae* (OTU0071), and *Staphylococcus epidermidis* (OTU0068), were present at comparatively low abundance.

### Taxonomy-based functional profiling of bacterial communities

**Figure 3:**
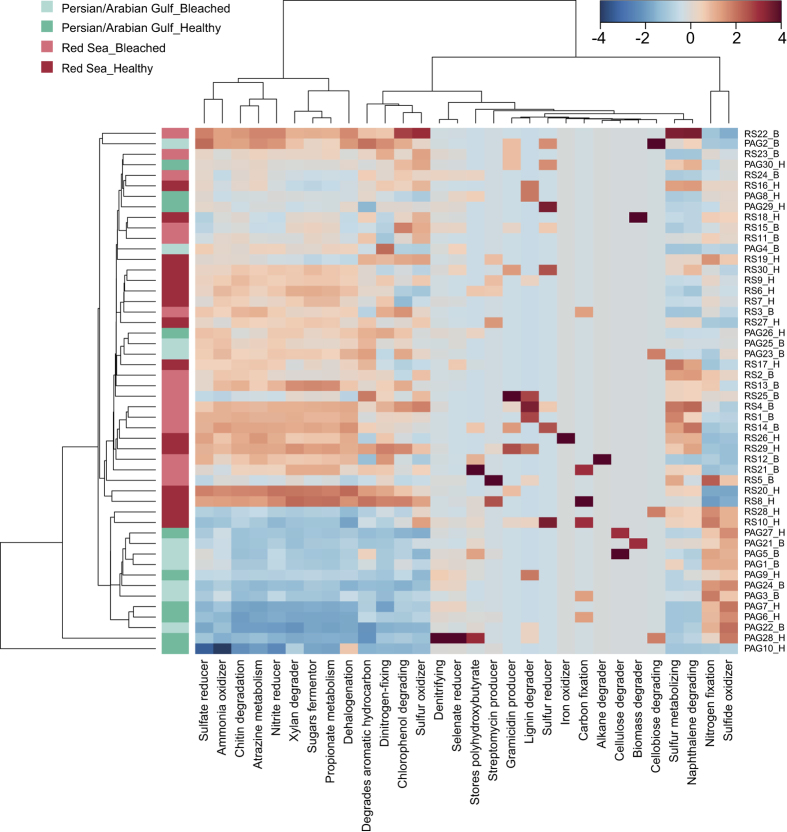
Taxonomy-based functional profiling of bacterial communities associated with mucus from *P. lobata* from the Persian/Arabian Gulf and Red Sea. The heatmap displays putative changes in bacterial community function. Changes are displayed on a relative scale with enrichment in red and depletion in blue. Rightmost column indicates sample names and health status (B: Bleached, H: Healthy).

To gain insight into putative functional differences associated with bacterial community differences, we applied taxonomy-based functional profiling (Fig. 3). The majority of samples grouped by region, although some samples from the PAG clustered with samples from the RS (Fig. 3). Within clustered samples from the PAG, the functions ‘Sulfate reducer’, ‘Ammonia oxidizer’, ‘Chitin degradation’, ‘Nitrite reducer’, ‘Xylan degrader’, ‘Sugar fermentor’, and ‘Dinitrogen-fixing’ were all less abundant in comparison to the RS where these processes were more abundant. In contrast, ‘Nitrogen fixation’ and ‘Sulfide oxidizer’ were abundant in the PAG and either unchanged or scarce in the RS (Fig. 3). To confirm an enrichment of ‘Dinitrogen-fixing’ in the RS, we assessed the abundance of diazotroph communities via qPCR of the nifH gene and could confirm an on average higher abundance of diazotrophs in samples from the RS in comparison to the PAG (27% increase; Supplementary Fig. S3), but this difference was not statistically significant (*P*_T-test_ ≥ 0.05).

## Discussion

In this study, we compared bacterial community composition of the SML from bleached and healthy coral colonies of *P. lobata* from the PAG and RS in order to determine their structure, stability, and putative functional profiles. Our results show that bacterial community composition of coral mucus is highly similar between bleached and healthy corals. Importantly, core bacterial microbiome members are comprised of abundant and rare bacterial associates, whereas site-specific and health state differences exist for less abundant bacteria. Further, algal symbiont association shows a similar pattern to bacterial community patterns, as we did not find differences between health states, but regional differences. For instance, we could confirm the presence of *Symbiodinium* type C3 in *P. lobata* from the PAG^43^,^44^,^45^, whereas *P. lobata* from the RS were associated with *Symbiodinium* types C15 (and variants thereof) as well as types from clade D^43^,^46^. However, we did not find apparent differences between bleached and healthy colonies. Hence, it remains to be seen whether a causal relationship between *Symbiodinium* and bacterial community patterns exists. Given that corals from both the PAG and RS are able to survive seasonal temperature maxima exceeding those form other regions, at least in part due to harboring algal thermal tolerant symbionts that are commonly associated with high temperature environments^44^,^45^,^47^, it would be intriguing to find bacterial associates that co-occur with symbiont types. In a recent study with the coral model *Aiptasia*, microbial community patterns were distinct between symbiotic and aposymbiotic anemones, arguing for a connection between bacterial community composition and the cnidarian-algal symbiosis^48^. At large, the presence of photosymbionts distinguishes the microbiomes of hosts from those without photosymbionts^49^, but microbiomes of juvenile corals hosting different *Symbiodinium* clades were indistinguishable^50^.

To our knowledge, this is the first study that compares mucus-associated bacteria from bleached and healthy *P. lobata* colonies. *Porites* spp. have high production rates of mucus that cover coral colonies in the form of mucus sheets that exhibit a distinct ageing cycle making it an ideal model system to study dynamics of the mucus-associated microbiota^20^,^23^. Commonly, a new fluid mucus layer is produced about every four weeks^20^,^23^. The microbiome of coral surface mucus has an important role in mediating holobiont health^20^, and hence, the periodical release of mucus supposedly supports maintaining a beneficial bacterial microbiome via the removal of undesirable bacteria from the coral colony surface. Notably, mucus surfaces in many domains of multicellular life provide a protective barrier function and are assumed to constitute a common organismal feature^51^. This is supported by our data, as we find that mucus-associated bacteria in bleached *P. lobata* are similar to those in healthy *P. lobata* colonies, and long-term monitoring of their post-bleaching survival rates could further corroborate this assumption. In contrast, Bourne, *et al*.^32^ showed a shift of bacteria associated with the tissue of bleached corals. This indicates that mucus-associated bacterial communities may be less dynamic than those that are tissue-associated. Accordingly, the stably associated bacteria in the SML may provide a protective function, even and especially when coral health is compromised as during coral bleaching. In this regard, Lee, *et al*.^52^ showed that under heat stress the chemical composition of mucus in the coral *Acropora muricata* changed, which might either influence the associated bacterial community or be a consequence of it. Based on the presence of stable bacterial associates in our study, we infer that the mucus chemical composition did not change, although coral bleaching likely affected the availability of carbohydrates for mucus production^23^.

It is interesting to note that all mucus samples were dominated by few OTUs that were previously reported from saline environments, arguing that the high salinity of the PAG and RS might indeed comprise a structural determinant of bacteria associated with corals in these regions. Further, the consistent presence of these taxa in all coral mucus samples irrespective of site or bleaching state implies that they play an important role in the coral holobiont. *Pseudomonas veronii* has previously been found in fungiid corals experimentally exposed to high salinities (49 PSU)^41^. *Dietzia* sp. is found in the marine environment and in soil, human skin, and the intestinal tract of a carp, and plays a role in biodegradation, bioremediation, industrial fermentation, and carotenoid pigmentation^53^. The presence of *Brachybacterium* sp. has previously been reported in oil-contaminated coastal sand^54^ and salt-fermented seafood^55^. In contrast, site-specific bacterial taxa displayed lower abundance on average. Nevertheless, these bacteria suggest that *Porites* spp. have the ability to harbor flexible, and presumably locally adjusted microbiomes, which might at least in part contribute to the resilience of this coral genus^56^,^57^.

Predictive bacterial functional profiling between the PAG and the RS revealed differences in the abundance of bacteria associated with sulfur and nitrogen cycling. Differences in sulfur cycling included a scarcity of ‘Sulfur oxidizer’ and ‘Sulfate reducer’ and an increased abundance of ‘Sulfide oxidizer’ in samples from the PAG. Corals and especially their endosymbiotic algae are major producers of dimethylsulfoniopropionate (DMSP)^58^. Its breakdown products, such as dimethyl sulfoxide (DMSO), result mainly from bacterial metabolism and play a significant role in the scavenging of harmful reactive oxygen species (ROS)^59^. Importantly, *Symbiodinium* produce elevated levels of ROS during thermal stress, which may result in coral bleaching^60^,^61^. Consequently, the high temperatures in the PAG likely triggers increased ROS production demanding increased availability of ROS scavengers such as DMSP and DMSO, which could explain the functional differences in sulfur cycling observed in SML-associated bacteria between the PAG and the RS.

Differences in nitrogen cycling included increased abundance of ‘Dinitrogen-fixing’, ‘Ammonia oxidizer’, and ‘Nitrite reducer’ in the RS. Efficient nitrogen fixation and nitrogen recycling is essential for corals to thrive in nutrient-limited environments^62^. The RS constitutes a highly oligotrophic environment where nitrogen is presumably not readily available for corals. Compared to the RS, nitrogen is not a limiting nutrient in the PAG^63^, allowing for comparably high uptake for nitrogen sources (increased abundance of ‘Nitrogen fixation’)^64^ and lacking the need for efficient nitrogen recycling (decreased abundance of ‘Ammonia oxidizer’). From our analyses, functional profiling supports that environmental conditions strongly influence bacterial nitrogen fixation in corals^65^.

Taken together, in this study we found stable bacterial communities in the SML of bleached and healthy coral colonies of *P. lobata* from the PAG and the RS. This underscores the barrier function of coral mucus and we argue that bleached coral colonies benefit from the stable composition and distribution of SML-associated bacteria that presumably provide protective functions. In line with this, we found several abundant and ubiquitous bacterial taxa that we identified as core bacterial microbiome members of coral mucus. Further, regional differences in the mucus bacterial microbiome between PAG and RS were represented by less abundant bacteria that could be associated with a shift in predicted bacterial functional profiles. The specific regional bacterial taxa may thus contribute to the success of *P. lobata* colonies across a range of environmental conditions.

## Methods

### Study sites and coral mucus collection

Coral mucus was collected from bleached and healthy *Porites lobata* colonies from two reefs in the PAG, Saadiyat (24°35′56.4″N 54°25′17.4″E; samples: PAG1–PAG10) and Ras Ghanada (24°50′53.2″N 54°41′25.1″E; samples: PAG21–PAG30), and from three reefs in the central RS, Shib Nazar (22°20′27.4″N 38°51′07.6″E; samples: RS1–RS10), Al-Fahal (22°15′06.0″N 38°57′23.2″E; RS11–RS20), and Inner Fsar (22°13′58.4″N 39°01′45.6″E; samples: RS21–RS30), in September and October 2012, respectively. For each reef, mucus from 5 bleached and 5 healthy coral colonies were collected at 6 to 8 m depth, comprising a total of 20 samples from the PAG and 30 samples from the RS. Following^66^, corals were considered bleached if at least 20% of the colony surface had lost coloration. Mucus samples were collected using sterile syringes by sucking up mucus from the coral surface and by irritating the surface with the syringe tip while concomitantly collecting the released mucus. Syringes were placed in sterile Whirl-Paks. Upon return to the boat, syringes were placed upside-down in order for the heavier mucus to settle. Water on top of mucus was discarded and remaining mucus was ejected into cryotubes and frozen in liquid nitrogen. Samples were stored at −80 °C. In addition to the mucus samples, 1 L of seawater from each reef was collected at 1 to 2 m depth with a cubitainer. Cubitainers were transported on ice and 500 ml of collected water samples were subsequently filtered on 0.22 μm Milipore Durapore filters (Millipore, Billerica, MA, USA). Filters were snap-frozen in liquid nitrogen and stored at −80 °C.

### DNA extraction

100 μL of mucus were used for DNA extraction using the Qiagen AllPrep DNA/RNA Mini kit (Qiagen, Hilden, Germany) according to the manufacturer’s protocol. For extraction of DNA from seawater filters, half of each filter was cut into small stripes with sterile razorblades and transferred into 2 ml test tubes. After adding 400 μL Qiagen RLT buffer, the samples were incubated on a rotating wheel for 20 minutes. Subsequent extraction steps were performed according to the manufacturer’s protocol. DNA concentrations were quantified on a NanoDrop 2000C spectrophotometer (Thermo Fisher Scientific, Waltham, MA, USA).

### Identification of algal symbionts from mucus of bleached and healthy *Porites* colonies

To determine algal symbionts, the ITS2 rDNA region was amplified with primers ITSintfor2 and ITS2CLAMP following^67^ with the following modifications: during touch-down PCR amplification the annealing temperature was decreased by 0.5 °C every cycle for 20 cycles, followed by 27 cycles at a final annealing temperature of 52 °C. *Symbiodinium* types were determined by DGGE profiling and subsequent sequencing of prominent bands. Prominent bands were excised from the DGGE gel and re-amplified as described in ref. 68. PCR products were then purified with Illustra ExoStar enzyme mix (SelectScience, Bath, UK), and samples were sequenced bidirectionally at the KAUST BioScience Core Laboratory (Thuwal, Saudi Arabia). Sequences were quality trimmed in CodonCode Aligner (CodonCode Corporation, Centerville, MA). Forward and reverse sequences were assembled into contigs and aligned using ClustalW. Each contig was BLASTed against a local reference database of *Symbiodinium* ITS2 sequences^69^ and against ITS2 sequences of type C15 variants recently described from *Porites* spp. in the Red Sea^46^.

### 16S rRNA gene sequencing

DNA isolated from coral mucus and seawater was used for PCR amplification of a portion of the 16S rRNA gene. Five to 35 ng DNA from mucus samples and 1 to 3 ng DNA from seawater samples were used to amplify variable regions 5 and 6 of the 16S rRNA gene with the primers 784 F [5′-TCGTCGGCAGCGTCAGATGTGTATAAGAGACAGAGGATTAGATACCCTGGTA-3′] and 1061 R [5′-GTCTCGTGGGCTCGGAGATGTGTATAAGAGACAGCRRCACGAGCTGACGAC-3′]^70^, which have been shown to amplify well with coral DNA^71^. Illumina MiSeq adaptor overhangs (underlined above; Illumina, San Diego, CA, USA) were used for subsequent library indexing. All PCRs were run in triplicates per sample using Qiagen Multiplex PCR Kit with 0.2 μM of each primer and a total reaction volume of 25 μL. Cycling conditions were as follows: 95 °C for 15 min, followed by 30 cycles of 95 °C for 30 s, 55 °C for 90 s, 72 °C for 30 s, and a final extension cycle of 72 °C for 10 min. Successful amplification was checked via 1% agarose gel electrophoresis and sample triplicates were pooled in equimolar ratios. Pooled samples were cleaned with Agencourt AMPpure XP magnetic beads system (Beckman Coulter, Brea, CA, USA) and subsequently underwent an indexing PCR to add Nextera XT indexing and sequencing adapters (Illumina) following the manufacturer’s protocol. PCR products were sequenced on the Illumina MiSeq platform at the KAUST BioScience Core Laboratory. Sequence data determined in this study are available under NCBI’s BioProject ID PRJNA352338, accessible at https://www.ncbi.nlm.nih.gov/bioproject/PRJNA352338/.

### Bacterial community analysis

Raw sequencing data were analyzed using mothur v.1.36.3^72^. Sequence reads were split according to barcodes, assembled to contigs, and quality trimmed. Identical sequences were merged using the ‘unique.seqs’ command to save computation time, and the command ‘count.seqs’ was used to keep a count of the number of sequences over samples represented by the remaining representative sequence. Sequences that occurred only once across the entire dataset were removed. The remaining sequences were aligned against SILVA database release 119^73^ and pre-clustered (3-bp difference)^74^. Chimeric sequences were removed using UCHIME^75^. Mitochondria, chloroplast, archaea, eukaryote, and unknown sequences were removed. Sequences were classified with the Greengenes database^76^ using a 60% bootstrap cut-off. For subsequent OTU (Operational Taxonomic Unit)-based analyses, samples were subsampled to 2,827 sequences as determined by the sample with the lowest number of sequences; a 97% similarity cut-off was then applied to obtain OTUs. Chao1 index, Simpson evenness, and Inverse Simpson Index were calculated as implemented in mothur. To assess differences between bacterial communities associated with mucus and seawater, analysis of MOlecular VAriance (AMOVA) was performed in mothur. AMOVA was further used to test for differences in bacterial communities between bleached and healthy colonies per region and for differences between regions. To determine OTUs that were associated with bleached and healthy colonies of *P. lobata* from either the PAG or the RS (and combinations thereof), the statistical package IndicSpecies^77^ was used using OTU abundances with a significance threshold of *P* ≤ 0.01. Due to the design of the IndicSpecies analysis, the identified bacterial OTUs are exclusive to the specific health state ∗ region combination tested.

For determination of the core bacterial microbiome, all OTUs were considered that were presented in >75% of all coral samples based on OTU abundance counts. Notably, more abundant taxa are potentially more likely to be consistently identified across samples, hence, the choice of the >75% cutoff. We categorized mucus core OTUs as ‘exclusive’ when they were not present in any of the water samples, as ‘mucus-dominant’ when the ratio of mean sequence counts in mucus over water samples was >1, and as ‘environmental’ when the ratio of mean sequence counts in mucus over water samples was <1.

Functional differences based on bacterial 16S community composition (OTU taxonomy and abundance), were assessed with METAGENassist^78^. Input files were created in mothur using the ‘make.shared’ and ‘classify.otu’ commands based on all coral samples. 1,978 distinct OTUs were assigned, mapped, condensed into 500 functional taxa, and filtered based on interquantile range^79^. After filtering, 375 functional taxa remained and were normalized over samples by sum and over taxa by Pareto scaling. These data were analyzed for ‘metabolism by phenotype’, and Euclidean distance measure (single clustering algorithm) was used to visualize the results in a heatmap.

### Relative abundance of nifH and 16S rRNA genes using qPCR

To confirm the increased functional abundance of ‘Dinitrogen-fixing’ in coral colonies from the RS (as inferred from METAGENassist), abundance of the nifH gene relative to abundance of the 16S rRNA gene was measured using quantitative PCR (qPCR). Reactions were run in triplicate per mucus sample on a 7900HT Fast Real-Time PCR System (Applied Biosystems, USA) using a reaction volume of 20 μL containing 2 μL of DNA (approximately 1 ng), 10 μL of Platinum SYBR green qPCR Supermix-UDG (Invitrogen, USA), and 0.4 μL of ROX reference dye. For amplification of the nifH gene, the primers F2 and R6^80^ were used; for amplification of the 16S rRNA gene, primers 784F and 1061R were used^70^. Amplification reactions were performed with a primer concentration of 0.2 μM and with an initial polymerase activation step at 50 °C for 2 min and a denaturation step at 94 °C for 1 min followed by 50 cycles of 94 °C for 30 sec, 51 °C for 60 s, 72 °C for 60 s, and a final step of 72 °C for 3 min with a subsequent melting curve analysis. Triplicate cycle threshold (Ct) values for each sample were averaged. Relative abundance differences based on differences in gene copy numbers were calculated using the equation ΔCt = (Ct_nifH_ − Ct_16S_) for all mucus samples from the PAG and RS. Fold-change (FC) difference of nifH between PAG and RS was calculated as FC = 2^−ΔΔCt^ with ΔΔCt = (ΔCt_RS_ − ΔCt_PAG_) and tested for differences between regions using a T-test. Efficiency of qPCR was 82.12% based on the formula E = 10^(−1/slope)^ − 1^81^ and the R^2^ of the standard curve was >0.99.

## Additional Information

**Accession codes**: Sequence data determined in this study are available under NCBI’s BioProject ID PRJNA352338, accessible at https://www.ncbi.nlm.nih.gov/bioproject/PRJNA352338/. Bacterial core microbiome OTU reference sequences are available under GenBank Accession numbers KY565430-KY565470.

**How to cite this article:** Hadaidi, G. *et al*. Stable mucus-associated bacterial communities in bleached and healthy corals of *Porites lobata* from the Arabian Seas. *Sci. Rep.* **7**, 45362; doi: 10.1038/srep45362 (2017).

**Publisher's note:** Springer Nature remains neutral with regard to jurisdictional claims in published maps and institutional affiliations.

## Supplementary Material

Supplementary Information

Supplementary Dataset S1

## Figures and Tables

**Table 1:** Summary statistics of 16S rRNA gene sequencing of mucus-associated bacteria from bleached and healthy coral colonies of *P. lobata* from the Persian/Arabian Gulf (PAG) and the Red Sea (RS).

Region	Condition	No. of Seqs	No. of OTUs*	Chao1*	Inverse Simpson*	Simpson evenness*
PAG reef 1	Bleached	37,634 (23,659)	171.2 (41.5)	218.4 (72.3)	17.6 (9.7)	0.10 (0.04)
PAG reef 1	Healthy	62,224 (43,410)	202.8 (18.1)	280.2 (48.7)	24.3 (13.2)	0.12 (0.06)
PAG reef 2	Bleached	51,272 (28,950)	188.8 (57.4)	259.2 (110.4)	21.4 (12.9)	0.11 (0.05)
PAG reef 2	Healthy	47,882 (32,597)	176.2 (33.1)	224.2 (76.1)	17.9 (10.0)	0.10 (0.04)
RS reef 1	Bleached	25,079 (18,013)	141.2 (34.4)	168.3 (48.0)	8.2 (3.5)	0.07 (0.01)
RS reef 1	Healthy	32,027 (19,659)	134.8 (42.5)	162.1 (56.6)	10.5 (7.2)	0.07 (0.02)
RS reef 2	Bleached	22,450 (15,270)	139.6 (39.2)	168.9 (57.2)	8.5 (3.3)	0.06 (0.01)
RS reef 2	Healthy	18,881 (12,127)	139.4 (44.5)	165.6 (56.7)	9.1 (3.7)	0.07 (0.01)
RS reef 3	Bleached	11,302 (5,581)	127.8 (24.9)	151.4 (24.8)	8.7 (3.3)	0.07 (0.02)
RS reef 3	Healthy	20,426 (10,672)	127.0 (24.6)	176.7 (36.7)	8.2 (4.6)	0.06 (0.02)
PAG reef 1	Water	207,396	266	570	36.2	0.14
PAG reef 2	Water	141,076	217	462	30.3	0.14
RS reef 1	Water	271,394	208	373	25.2	0.12
RS reef 2	Water	250,718	228	403	24.9	0.11
RS reef 3	Water	324,309	184	395	25.8	0.14

Numbers are provided as means and standard deviation (SD); for corals n = 5, for water n = 1.

^*^Subsampled to 2,827 sequences; PAG reef 1: Saadiyat; PAG reef 2: Ras Ghanada; RS reef 1: Shib Nazaar; RS reef 2: Al Fahal reef; RS reef 3: Inner Fsar. Total number of OTUs: 2,225.

**Table 2:** Core bacterial microbiome of mucus-associated bacteria from bleached and healthy colonies of *P. lobata* from the PAG and the RS.

OTU ID	Pres.	Taxonomy	Mean Abundance (mucus)	Mean Abundance (water)
Otu0001	100%	*Pseudomonas veronii*^#^	729.8	2.0
Otu0002	100%	*Brachybacterium* sp.^#^	314.6	2.0
Otu0003	100%	*Dietzia* sp.^#^	204.7	1.4
Otu0004	100%	Unknown species, family OCS155^+^	87.3	256.0
Otu0005	100%	Unknown species, family Pelagibacteraceae^+^	61.5	207.8
Otu0008	100%	*Pseudomonas umsongensis* (91)^#^	53.1	0.2
Otu0011	100%	*Caulobacter henricii*^#^	44.2	0.4
Otu0012	100%	*Herbaspirillum* sp.*	43.3	n.d.
Otu0018	100%	Brevibacterium aureum*	30.8	n.d.
Otu0020	100%	*Pelomonas puraquae*^#^	27.5	0.2
Otu0029	100%	Sphingomonas echinoides*	17.5	n.d.
Otu0035	100%	*Acinetobacter guillouiae* (81)^#^	14.8	11.2
Otu0036	100%	*Propionibacterium acnes*^#^	14.7	2.8
Otu0016	98%	Unknown species, class Deltaproteobacteria^+^	25.0	68.0
Otu0019	98%	*Candidatus Portiera* sp.^+^	21.4	73.0
Otu0007	96%	*Candidatus Portiera* sp.^+^	45.4	118.6
Otu0009	96%	Unknown species, family Rhodospirillaceae^+^	38.6	111.2
Otu0021	96%	*ZA3312c* sp.^+^	21.5	57.2
Otu0040	96%	Unknown species, family Pelagibacteraceae^+^	10.6	37.8
Otu0006	94%	Unknown species, family Rhodobacteraceae^+^	69.4	116.0
Otu0023	94%	Unknown species, class Alphaproteobacteria^+^	19.5	59.0
Otu0015	92%	Unknown species, family Flammeovirgaceae^+^	25.8	91.2
Otu0032	92%	Unknown species, family Pelagibacteraceae^#^	12.9	0.4
Otu0037	92%	*Phaeobacter* sp. (99)^+^	14.8	39.6
Otu0013	90%	Unknown species, family Flavobacteriaceae^+^	31.5	52.6
Otu0017	90%	Unknown species, family Flavobacteriaceae^+^	23.6	75.2
Otu0027	90%	*Polaribacter irgensii* (91)^+^	16.4	33.6
Otu0051	90%	Unknown species, family Pelagibacteraceae^+^	6.0	20.0
Otu0033	88%	Unknown species, order Rhizobiales^+^	13.2	29.2
Otu0063	88%	*Delftia* sp.*	6.4	n.d.
Otu0010	86%	*Candidatus_Portiera* sp.^+^	35.5	111.0
Otu0030	86%	Unknown species, family OM60^+^	14.3	29.2
Otu0071	86%	Sphingobium yanoikuyae*	5.1	n.d.
Otu0050	84%	Unknown species, order MWH-UniP1^+^	6.4	17.0
Otu0043	82%	Unknown species, family Piscirickettsiaceae^+^	8.6	27.6
Otu0025	80%	Unknown species, family Flavobacteriaceae^+^	21.9	28.0
Otu0041	80%	Unknown species, family Flavobacteriaceae^+^	11.9	22.2
Otu0045	80%	Unknown species, family AEGEAN_112^+^	8.4	23.2
Otu0068	80%	Staphylococcus epidermidis*	5.8	n.d.
Otu0022	78%	*Polaribacter irgensii*^+^	22.8	31.6
Otu0044	78%	Unknown species, class Gammaproteobacteria^+^	8.9	19.4
Otu0114	76%	Unknown species, order Actinomycetales^#^	2.1	0.2

Core bacterial microbiome members are present in >75% of all coral mucus samples. Taxonomic classification of OTUs against Greengenes database (bootstrap value indicated if <100). Mean abundance denotes average number of sequence counts over all mucus or water samples, respectively.

Core bacterial microbiome OTUs categorized as follows: * = ‘exclusive’ (i.e., not present in water samples), ^#^ = ‘mucus-dominant’ (i.e., ratio of mean sequence counts in mucus over water samples >1), and ^+^ = ‘environmental’ (i.e., ratio of mean sequence counts in mucus over water samples <1). n.d. = not detected.
