# Supplementary material for: Stable mucus-associated bacterial communities in bleached and healthy corals of Porites lobata from the Arabian Seas
Source: Sci Rep. 2017 Mar 31;7:45362. doi: 10.1038/srep45362 (PMC5374439; doi:10.1038/srep45362)
Supplement: Supplementary Information [file srep45362-s1.pdf]

Supplementary Information

Stable mucus-associated bacterial communities in bleached and healthy corals of *Porites lobata* from the Arabian Seas

Ghaida Hadaidi[1], Till Röthig[1], Lauren K. Yum[1], Maren Ziegler[1], Chatchanit Arif[1], Cornelia Roder[1], John Burt[2], Christian R. Voolstra[1,*]

[1]Red Sea Research Center, Division of Biological and Environmental Science and Engineering (BESE), King Abdullah University of Science and Technology (KAUST), Saudi Arabia

[2]Center for Genomics and Systems Biology, New York University Abu Dhabi, PO Box 129188, Abu Dhabi, United Arab Emirates

*Corresponding author, Contact information:
Email: christian.voolstra@kaust.edu.sa
Tel: +966 12 8082377, Fax: +966 21 8082377

Running title: Stable bacterial communities of bleached and healthy coral

Keywords: coral reef, ecosystem, bacterial community profiling, 16S rRNA gene, Red Sea, Persian/Arabian Gulf, coral bleaching, nifH gene

Supplementary Tables

Supplementary Table S1. *Symbiodinium* types associated with bleached and healthy *Porites lobata* from the Persian/Arabian Gulf (PAG) and the Red Sea (RS) using DGGE profiling of ITS2. Samples PAG1 – PAG10: Saadiyat reef; PAG21 – PAG30: Ras Ghanada reef; RS1 – RS10: Shib Nazaar reef; RS11 – RS20: Al Fahal reef; RS21 – RS30: Inner Fsar reef. ND = not determined due to difficulties in amplifying *Symbiodinium* DNA from coral mucus samples.

Sample name	Condition	*Symbiodinium* type	Identity to ITS2 type sequence (%)
PAG1	Bleached	ND	ND
PAG2	Bleached	ND	ND
PAG3	Bleached	ND	ND
PAG4	Bleached	ND	ND
PAG5	Bleached	ND	ND
PAG6	Healthy	C3	100
PAG7	Healthy	C3	100
PAG8	Healthy	C3	100
PAG9	Healthy	C3	100
PAG10	Healthy	C3	100
PAG21	Bleached	ND	ND
PAG22	Bleached	ND	ND
PAG23	Bleached	ND	ND
PAG24	Bleached	ND	ND
PAG25	Bleached	ND	ND
PAG26	Healthy	C3	100
PAG27	Healthy	C3	100
PAG28	Healthy	C3	100
PAG29	Healthy	C3	100
PAG30	Healthy	C3	100
RS1	Bleached	ND	ND
RS2	Bleached	A1	99
RS3	Bleached	C15h	100
RS4	Bleached	ND	ND
RS5	Bleached	C97	100
RS6	Healthy	D1	99
RS7	Healthy	C15n	100
RS8	Healthy	D1	100
RS9	Healthy	ND	ND

RS10	Healthy	D1a	100
RS11	Bleached	D1	100
RS12	Bleached	ND	ND
RS13	Bleached	C15	100
RS14	Bleached	ND	ND
RS15	Bleached	C15	100
RS16	Healthy	D1	100
RS17	Healthy	C15n	100
RS18	Healthy	ND	ND
RS19	Healthy	ND	ND
RS20	Healthy	C15n	100
RS21	Bleached	D1	100
RS22	Bleached	ND	ND
RS23	Bleached	D1	100
RS24	Bleached	C15n	100
RS25	Bleached	C15/D1/D1a	100/100/100
RS26	Healthy	C15n	100
RS27	Healthy	C15n	100
RS28	Healthy	D1/D6	100/100
RS29	Healthy	C15n	100
RS30	Healthy	C15n	99

Supplementary Table S2. Summary statistics of 16S rRNA gene sequencing of mucus-associated bacteria from bleached and healthy coral colonies of *P. lobata* from the Persian/Arabian Gulf (PAG) and the Red Sea (RS) (full dataset: 50 coral and 5 water samples).

Sample name	Condition	No. of Seqs	No. of OTUs*	Chao1*	Inverse Simpson*	Simpson evenness*
PAG1	Bleached	42,715	178	199	22.1	0.12
PAG2	Bleached	12,468	122	148	4.7	0.04
PAG3	Bleached	67,164	209	298	25.1	0.12
PAG4	Bleached	14,702	135	157	9.7	0.07
PAG5	Bleached	51,122	212	291	26.2	0.12
PAG6	Healthy	93,539	226	331	39.2	0.17
PAG7	Healthy	109,719	215	307	37.6	0.18
PAG8	Healthy	25,004	182	215	12.9	0.07
PAG9	Healthy	9,404	189	243	19.9	0.11
PAG10	Healthy	73,454	202	306	12.1	0.06
PAG21	Bleached	48,911	176	236	23.0	0.13
PAG22	Bleached	101,940	209	312	34.9	0.17
PAG23	Bleached	35,099	140	165	7.8	0.06
PAG24	Bleached	36,141	278	423	32.6	0.12
PAG25	Bleached	34,270	141	160	8.5	0.06
PAG26	Healthy	22,493	147	164	10.4	0.07
PAG27	Healthy	48,223	198	244	25.4	0.13
PAG28	Healthy	101,028	223	349	31.6	0.14
PAG29	Healthy	47,732	163	180	12.5	0.08
PAG30	Healthy	19,933	150	183	9.7	0.06
RS1	Bleached	3,526	116	120	5.9	0.05
RS2	Bleached	17,421	147	199	8.3	0.06
RS3	Bleached	19,910	107	127	6.2	0.06
RS4	Bleached	33,414	141	163	6.3	0.05
RS5	Bleached	51,126	195	233	14.1	0.07
RS6	Healthy	13,414	124	144	7.8	0.06
RS7	Healthy	38,949	128	150	9.3	0.07
RS8	Healthy	18,131	89	105	4.9	0.06
RS9	Healthy	27,057	128	155	7.5	0.06
RS10	Healthy	62,584	205	257	23.1	0.11
RS11	Bleached	39,941	145	211	10.4	0.07
RS12	Bleached	16,671	143	161	6.5	0.05
RS13	Bleached	3,458	99	105	6.9	0.07
RS14	Bleached	15,935	111	125	5.3	0.05

RS15	Bleached	36,244	200	242	13.4	0.07
RS16	Healthy	38,669	145	168	9.7	0.07
RS17	Healthy	19,028	149	176	7.1	0.05
RS18	Healthy	9,036	157	200	12.7	0.08
RS19	Healthy	18,782	182	214	12.1	0.07
RS20	Healthy	8,892	64	70	3.9	0.06
RS21	Bleached	15,590	148	165	7.7	0.05
RS22	Bleached	10,802	86	115	4.2	0.05
RS23	Bleached	17,046	143	168	8.8	0.06
RS24	Bleached	10,244	137	172	9.3	0.07
RS25	Bleached	2,827	125	136	13.3	0.11
RS26	Healthy	11,681	97	127	4.9	0.05
RS27	Healthy	12,887	127	145	7.5	0.06
RS28	Healthy	30,899	160	213	16.1	0.10
RS29	Healthy	33,222	111	126	5.3	0.05
RS30	Healthy	13,440	140	172	7.1	0.05
PAGW1	Seawater	207,396	266	570	36.2	0.14
PAGW2	Seawater	141,076	217	462	30.3	0.14
RSW1	Seawater	271,394	208	373	25.2	0.12
RSW2	Seawater	250,718	228	403	24.9	0.11
RSW3	Seawater	324,309	184	395	25.8	0.14

*subsampled to 2,827 sequences; Samples PAG1 - PAG10: Saadiyat reef; PAG21 - PAG30: Ras Ghanada reef; RS1 - RS10: Shib Nazaar reef; RS11 - RS20: Al Fahal reef; RS21 - RS30: Inner Fsar reef. Total number of OTUs: 2,225.

Supplementary Table S3. Site-specific bacterial taxa associated with mucus samples of *P. lobata* from the Persian/Arabian Gulf or the Red Sea ranked by relative abundance (average number of sequence counts). Taxonomic classification of OTUs against Greengenes database (bootstrap value indicated if < 100), the association value indicates the strength of the association for the respective OTU with the tested sample group.

OTU ID	Assoc. Value	*P* value	Taxonomy	Mean seq. abundance (group)
**Group: Persian/Arabian Gulf**				
Otu0010	0.930	0.001	*Candidatus Portiera* sp.	71.95
Otu0024	0.993	0.001	Unknown species, family Flavobacteriaceae	55.10
Otu0026	0.996	0.001	Unknown species, family S25_1238	48.15
Otu0039	0.902	0.001	*Polaribacter irgensii*	27.30
Otu0046	0.939	0.001	Unknown species, family Cryomorphaceae	20.50
Otu0049	0.897	0.001	*Flavobacterium* sp. (94)	14.05
Otu0052	0.907	0.001	Unknown species, family Cryomorphaceae	13.15
Otu0053	0.919	0.001	Unknown species, class Gammaproteobacteria	13.75
Otu0054	1.000	0.001	Unknown species, family Flavobacteriaceae	17.75
Otu0057	1.000	0.001	*Balneola* sp.	16.45
Otu0059	0.893	0.001	*Acholeplasma* sp.	10.00
Otu0065	0.975	0.001	Unknown species, family Flavobacteriaceae	12.65
Otu0073	0.906	0.001	Unknown species, family C111	8.45
Otu0074	0.838	0.001	Unknown species, family Saprospiraceae	9.25
Otu0075	1.000	0.001	Unknown species, family Rhodospirillaceae	9.95
Otu0076	0.846	0.001	*HTCC* sp.	7.30
Otu0077	0.916	0.001	*Fluviicola* sp.	8.50
Otu0078	0.973	0.001	Unknown species, class Alphaproteobacteria	8.75
Otu0079	0.819	0.001	*Photobacterium angustum* (91)	8.55
Otu0082	0.877	0.001	Unknown species, family Flavobacteriaceae	7.20
Otu0083	0.924	0.001	Unknown species, class Alphaproteobacteria	7.45
Otu0087	0.943	0.001	Unknown species, family Cryomorphaceae	7.75
Otu0090	0.903	0.001	Unknown species, family Saprospiraceae	5.45
Otu0091	0.833	0.001	Unknown species, family Cryomorphaceae	5.75
Otu0092	0.671	0.001	Unknown species, kingdom Bacteria	8.35
Otu0096	0.894	0.001	*Acholeplasma* sp.	6.05
Otu0104	0.784	0.001	*Candidatus_Portiera* sp.	4.25
Otu0105	0.811	0.001	Unknown species, class Alphaproteobacteria	4.75
Otu0109	0.922	0.001	Unknown species, order Kiloniellales	5.00
Otu0112	0.956	0.001	Unknown species, family Rhodobacteraceae	4.95

Otu0126	0.500	0.01	Unknown species, kingdom Bacteria	3.90
Otu0128	0.644	0.002	Unknown species, order Rhizobiales	1.95
Otu0131	0.775	0.001	Unknown species, family Saprospiraceae	3.05
Otu0141	0.832	0.001	Unknown species, family OM60	2.75
Otu0144	0.839	0.001	Unknown species, family Cryomorphaceae	2.50
Otu0145	0.500	0.009	*Wandonia* sp.	3.10
Otu0148	0.742	0.001	Unknown species, family Microbacteriaceae	2.90
Otu0149	0.811	0.001	Unknown species, family OM27	2.10
Otu0150	0.775	0.001	Unknown species, family Methylophilaceae	2.20
Otu0152	0.773	0.001	Unknown species, order GMD14H09	2.30
Otu0163	0.707	0.001	Unknown species, family Flavobacteriaceae	2.25
Otu0165	0.548	0.002	*Flammeovirga* sp.	2.45
Otu0171	0.721	0.001	Unknown species, family Rhodospirillaceae	1.50
Otu0174	0.671	0.002	Unknown species, family Phyllobacteriaceae	1.70
Otu0176	0.626	0.001	Unknown species, family Saprospiraceae	1.65
Otu0196	0.632	0.001	Unknown species, order Spirobacillales	1.30
Otu0197	0.668	0.003	*Turneriella* sp.	1.40
Otu0206	0.742	0.001	Unknown species, order Flavobacteriales	1.65
Otu0207	0.697	0.001	Unknown species, class Alphaproteobacteria	1.10
Otu0209	0.671	0.001	Unknown species, family Flavobacteriaceae	1.35
Otu0211	0.689	0.001	*Turneriella* sp.	1.25
Otu0217	0.77	0.001	Unknown species, family Cryomorphaceae	1.10
Otu0240	0.592	0.001	*Jannaschia* sp.	1.20
Otu0242	0.548	0.005	Unknown species, family Saprospiraceae	1.20
Otu0244	0.500	0.008	Unknown species, family Cryomorphaceae	1.20
Otu0247	0.632	0.001	Unknown species, order Rhizobiales	1.05
Otu0250	0.588	0.007	*Photobacterium damselae* (84)	0.85
Otu0258	0.548	0.004	Unknown species, order GMD14H09	1.00
Otu0261	0.500	0.01	Unknown species, class Alphaproteobacteria	1.10
Otu0262	0.632	0.001	Unknown species, class Gammaproteobacteria	0.75
Otu0275	0.632	0.001	Unknown species, family Saprospiraceae	0.80
Otu0292	0.500	0.009	Unknown species, family Flavobacteriaceae	0.85
Otu0300	0.500	0.008	*Saprospira* sp.	0.65
Otu0302	0.500	0.008	Unknown species, phylum Bacteroidetes	0.60
Otu0304	0.632	0.001	Unknown species, class Gammaproteobacteria	0.60
Otu0325	0.548	0.005	Unknown species, family Saprospiraceae	0.60
Otu0328	0.500	0.008	Unknown species, phylum Bacteroidetes	0.60
Otu0334	0.500	0.009	Unknown species, class Pedosphaerae	0.65
Otu0347	0.592	0.003	Unknown species, kingdom Bacteria	0.35
Otu0372	0.500	0.008	Unknown species, class Gammaproteobacteria	0.50
**Group: Red Sea**				
Otu0014	0.931	0.001	Unknown species, family OCS155	45.73
Otu0028	0.989	0.001	*Candidatus Portiera* sp.	23.73

Otu0034	0.966	0.001	*SGSH944* sp.	18.70
Otu0042	0.874	0.001	*Flavobacterium* sp.	15.10
Otu0056	0.852	0.001	*Candidatus_Portiera* sp.	8.77
Otu0080	0.730	0.002	Unknown species, kingdom Bacteria	5.10
Otu0081	0.707	0.001	Unknown species, family Flavobacteriaceae	5.73
Otu0085	0.775	0.001	*Candidatus Portiera* sp.	5.17
Otu0086	0.775	0.001	*Candidatus Portiera* sp.	5.10
Otu0088	0.787	0.001	Unknown species, family Rhodobacteraceae	4.70
Otu0089	0.730	0.001	Unknown species, family Flavobacteriaceae	4.87
Otu0097	0.742	0.003	Unknown species, family Endozoicimonaceae	4.23
Otu0110	0.775	0.001	*SGSH944* sp.	3.20
Otu0116	0.752	0.001	*Methylobacterium mesophilicum* (96)	3.10
Otu0125	0.683	0.001	Unknown species, class A712011	1.70
Otu0130	0.632	0.005	Unknown species, phylum Proteobacteria	1.90
Otu0134	0.614	0.007	Unknown species, family HTCC2089	1.63
Otu0140	0.658	0.003	Unknown species, class Alphaproteobacteria	1.53
Otu0142	0.632	0.002	*SargSea-WGS* sp.	1.77
Otu0143	0.753	0.001	Unknown species, family Pelagibacteraceae	1.60
Otu0159	0.577	0.009	*Candidatus Portiera* sp.	1.73
Otu0169	0.683	0.001	Unknown species, family Pelagibacteraceae	1.30
Otu0192	0.683	0.001	Unknown species, family Pelagibacteraceae	1.10
Otu0309	0.632	0.003	*Zhihengliuella* sp. (63)	0.53

Supplementary Table S4. Indicator bacterial taxa associated with bleached or healthy mucus samples of *P. lobata* from the Persian/Arabian Gulf or the Red Sea ranked by relative abundance (average number of sequence counts). Taxonomic classification of OTUs against Greengenes database ((bootstrap value indicated if < 100), the association value indicates the strength of the association for the respective OTU with the tested sample group.

OTU ID	Assoc. Value	*P* value	Taxonomy	Mean seq. abundance (group)
**Group: Persian/Arabian Gulf - bleached**				
Otu0328	0.606	0.004	unclassified Bacteroidetes (phylum)	1.1
Otu0378	0.581	0.005	*Vibrio ichthyoenteri*	0.9
Otu0481	0.548	0.009	unclassified OM27 (family)	0.4
**Group: Persian/Arabian Gulf - healthy**				
Otu0092	0.763	0.001	unclassified Bacteria (kingdom)	16.2
Otu0260	0.632	0.001	unclassified Gammaproteobacteria(class)	2.2
Otu0351	0.632	0.005	*Rickettsia* sp.	1.3
Otu0126	0.628	0.005	unclassified Bacteria (kingdom)	7.7
Otu0419	0.588	0.008	unclassified OM27 (family)	0.6
**Group: Red Sea - healthy**				
Otu0225	0.687	0.001	*Rhodococcus* sp.	1.6

Supplementary Figures

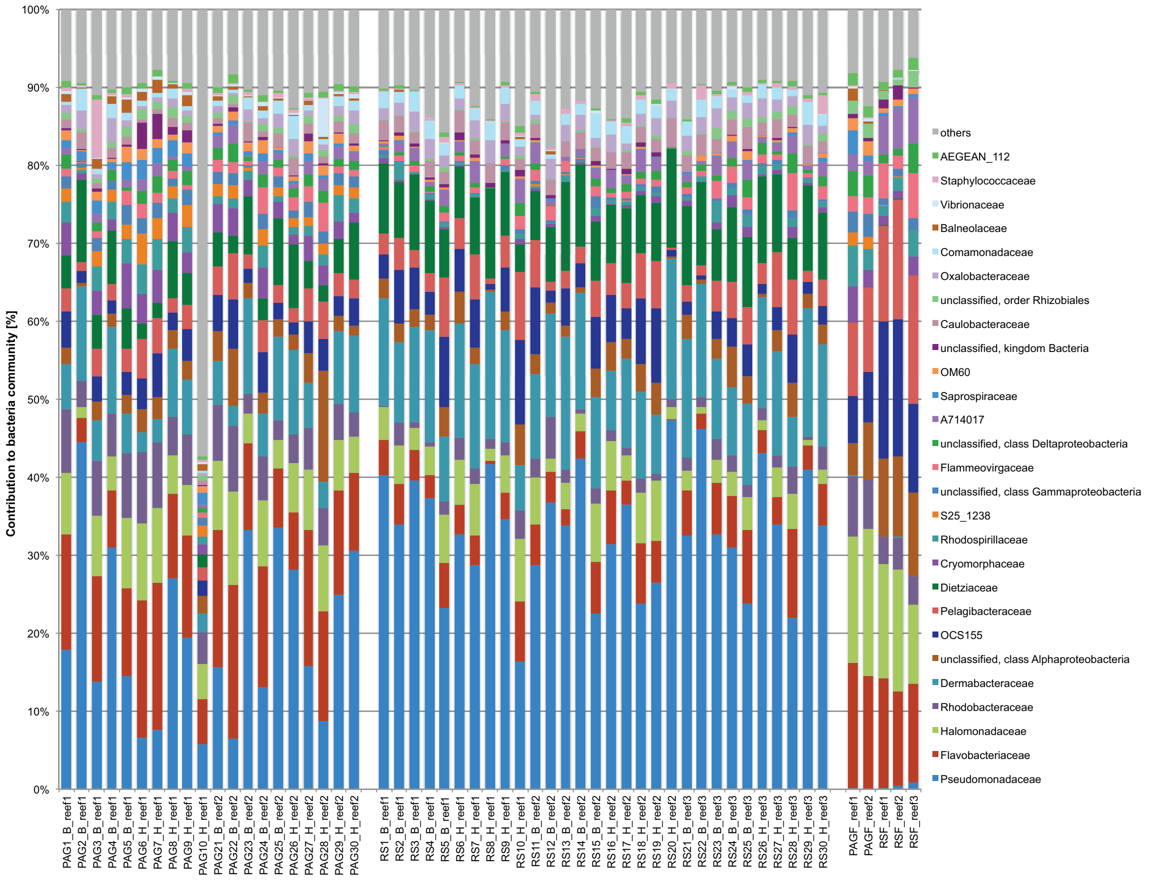

Supplementary Figure S1. Bacterial community composition of mucus from bleached and healthy coral colonies of *P. lobata* from the Persian/Arabian Gulf (PAG) and Red Sea (RS). Depicted is a taxonomy stacked column plot on the phylogenetic level of family. Each color represents one of the 27 most abundant families. Remaining taxa are grouped under category 'others'. Samples are ordered by site, reef, and health-state. B: Bleached; H: Healthy, Samples `PAG1 - PAG10: Saadiyat reef; PAG21 - PAG30: Ras Ghanada reef; RS1 - RS10: Shib Nazaar reef; RS11 - RS20: Al Fahal reef; RS21 - RS30: Inner Fsar reef.

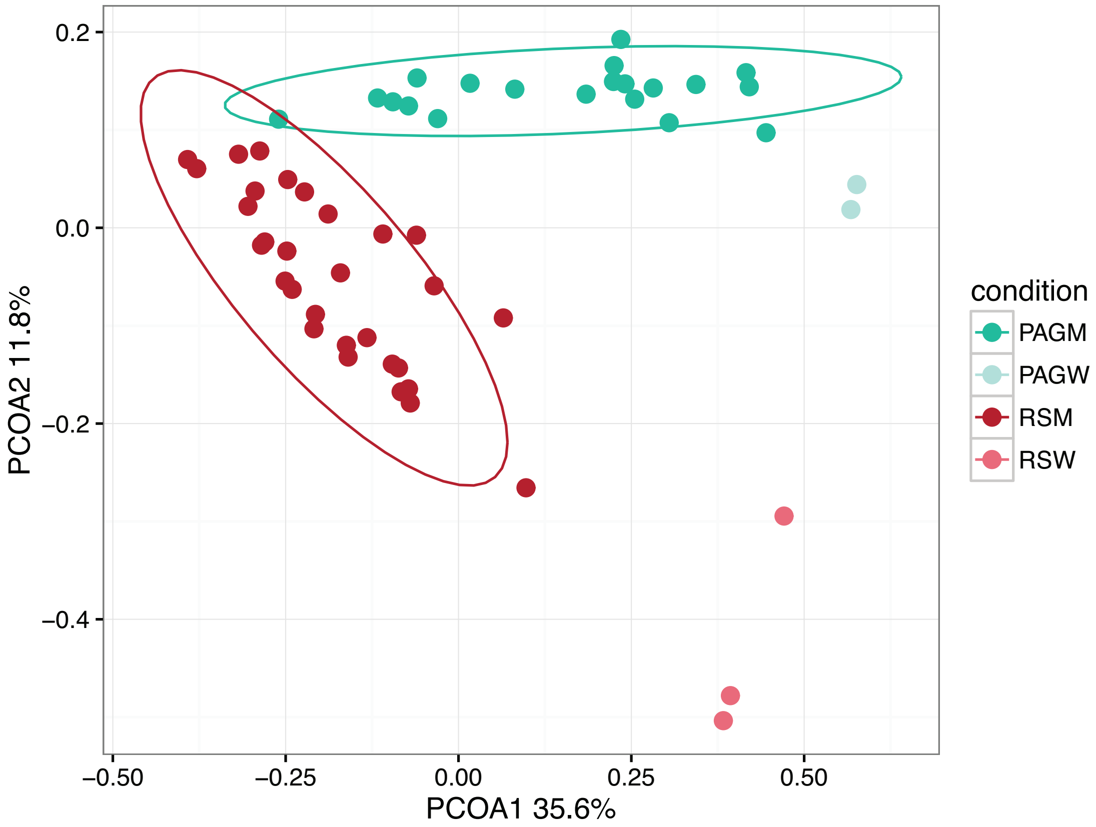

Supplementary Figure S2. Bacterial community composition from coral mucus of *Porites lobata* and water samples from the Persian/Arabian Gulf (PAG) and the Red Sea (RS). Principal coordinate analysis based on Operational Taxonomic Unit (OTU) abundance (sequence counts) shows differences between mucus and water samples, M: mucus samples; W: water samples. Ellipses denote 95 % confidence intervals per group.

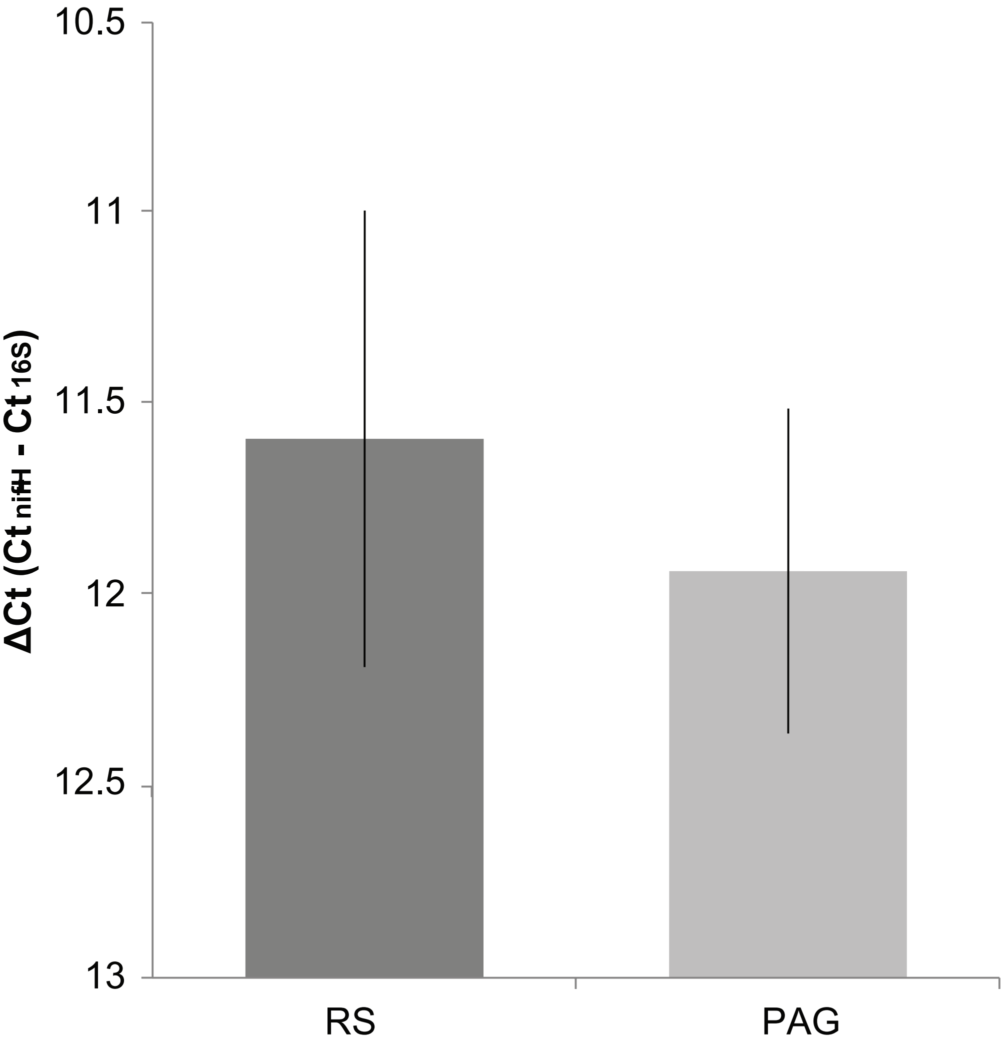

Supplementary Figure S3. Relative abundance of diazotrophs in mucus samples from *P. lobata* from the Red Sea (RS) and the Persian/Arabian Gulf (PAG). For ease of reference y-axis on reverse scale, lower values (= higher bars) indicate higher abundance; error bars = SE.

Supplementary Dataset S1. Overview over sequence counts, taxonomic classification, and 16S reference amplicon sequences for all OTUs identified.
